# High Throughput Virtual Screening to Identify Novel natural product Inhibitors for MethionyltRNA-Synthetase of Brucella melitensis

**DOI:** 10.6026/97320630013008

**Published:** 2017-01-20

**Authors:** Madhulata Kumari, Subhash Chandra, Neeraj Tiwari, Naidu Subbarao

**Affiliations:** 1 Department of Information Technology, Kumaun University, SSJ Campus, Almora, Uttarakhand 263601, India; 2 Department of Botany, Kumaun University, SSJ Campus, Almora, Uttarakhand 263601, India; 3 Department of Statistics, Kumaun University, SSJ Campus Almora, Uttarakhand, 263601, India; 4 School of Computational and Integrative Sciences, Jawaharlal Nehru University, New Delhi, 110067, India

**Keywords:** Brucella melitensis, Methionyl-tRNA-Synthetase, Molecular docking, HTVS

## Abstract

The Brucella melitensis methionyl-tRNA-synthetase (MetRSBm) is a promising target for brucellosis drug development. The virtual screening of large libraries of a drug like molecules against a protein target is a common strategy used to identify novel inhibitors. A High throughput virtual screening was performed to identify hits to the potential antibrucellosis drug target, MetRSBm. The best inhibitor identified from the literature survey was 1312, 1415, and 1430. In the virtual screening 56,400 compounds of ChEMBL antimycobacterial library, 1596 approved drugs, 419 Natural product IV library, and 2396 methionine analogous were docked and rescoring, identified top 10 ranked compounds as anti-mycobacterial leads showing G-scores -10.27 to -8.42 (in kcal/mol), approved drugs G-scores -9.08 to -6.60 (in kcal/mol), Natural product IV library G-scores -10.55 to -6.02 (in kcal/mol), methionine analogous Gscores -11.20 to -8.51 (in kcal/mol), and compared with all three known inhibitors (as control) G-scores -3.88 to -3.17 (in kcal/mol). This result indicates these novel compounds have the best binding affinity for MetRSBm. In this study, we extrapolate that the analogous of methionine for find novel drug likeness has been identified [4-(L-histidyl)-2-phenylbenzoyl] methionine hydrochloride, might show the inhibitor of Brucella melitensis effect by interacting with MetRS enzyme. We suggests that Prumycin as a natural product is the novel drugs for brucellosis.

## Background

Brucella spp. is a Gram-negative, nonencapsulated, flagellated, facultatively intracellular coccobacilli, causing of brucellosis, which is a zoonosis transmitted from animals to humans by ingestion of contaminated foods such as milk products, direct contact with an infected animal, or inhalation of aerosols . Four species of Brucella out of eight are known to cause disease; they are B. abortus, B. canis, B. suis, and B. melitensis which infect livestock and could also infect a human [1]. Brucella melitensis causes Ovine Brucellosis, along with Brucella ovis. It can infect sheep, cattle, and sometimes humans and transmitted by the stable fly, unlike Brucella ovis, causing Malta fever or localized brucellosis in humans.

The global burden of human brucellosis has been estimated more than 5 lakh human infections per year worldwide [2]. Brucella species have been reported to acquire antibiotic resistance resulting very difficult to treat. This bacteria could reside inside the host's cells and is able to envade the immune response and inhibit programmed cell death which provides it an extended life span [3,4]. The comparative efficacy of standard antibiotics on this intracellular pathogen and antibiotic resistance hamper successful treatment of the infection. Therefore combinations of the antibiotics: doxycycline, rifampin and streptomycin are applied in order to avoid relapses and to prevent prolonged use of these drugs include [5]. The therapeutic failures have been reported due to antibiotic resistance, which is associated with increasing prevalence of drug-resistance genes for the brucellosis first-line treatment options [6,7]. This situation demands to discover novel drug candidates to combat Brucella’s infection. In order to that researchers are applying molecular target based drug development. Among various molecular targets, methionyltRNA synthetase (MetRS) (generating increased interest from a drug development standpoint) is an excellent target for discovery of new drugs against Brucella. This protein is attracting very interest for drug development because it involves in cell protein translation processes [8]. MetRS is a novel target because it links tRNA with methionine for elongation in protein synthesis as well as with the initiator tRNA with methionine for protein synthesis [9]. In this study, we describe virtual screening of inhibitors of MetRS of B. melitensis (MetRSBm) by using the antimycobacterial library from ChEMBL Bioassay, approved drug dataset, Natural Products Set IV and Methionine analogous dataset. The novel potential inhibitors described in this research could be better as compared to the known inhibitors of MetRSBm.

## Methodology

### Identification of Positive Control

Positive control dataset consists of molecules identified for their inhibitory effect against Methionyl-tRNA-Synthetase enzyme from a survey of the literature. 2-({3-[(3,5-dichlorobenzyl) amino] propyl} amino)quinolin-4(1H)-one(1312), 1-{3-[(3-chloro-5- ethoxybenzyl) amino] propyl}- 3-phenylurea (1415), and 1-{3- [(3,5-dichlorobenzyl)amino] propyl} -3- thiophen-3-ylurea] (1430) [10], are present in positive control library.

### Datasets for High throughput virtual screening

A library of 56,400 compounds was obtained from the ChEMBL antimycobacterial database used for finding novel inhibitors against MetRSBm [https://www.ebi.ac.uk/chembl/]. An approved drug dataset containing 1596 compounds was extracted from drug bank (http://www.drugbank.ca/), The Natural Products Set IV consists of 419 compounds that were selected from the DTP Open Repository collection of 140,000 compounds. Factors in selection were origin, purity, structural diversity and availability of compound. (https://wiki.nci.nih.gov/display/NCIDTPdata/Compound+Se ts), and 2396 Methionine analogous were extracted from NCBI PubChem (pubchem.ncbi.nlm.nih.gov). Molecule Preprocessing of ligand molecules involved the conversion of dimension from 2D to 3D and conversion of file format to .sdf using Corina 2.64v software [11].

### Docking and Scoring

Molecular docking was performed using GLIDE module of Schrödinger Maestro, Version 9.1 [12] using against the MetRsBm. Adding hydrogen and generating conformations through the LigPrep module first prepared the ligand libraries. This LigPrep module generated tautomer with the OPLS2005 force field. The total no. of 141476, 2110, 5276, and 5782 output structures were obtained from ChEMBL antimycobacterial dataset, approved drugs, Natural product IV, and Methionine analogus dataset respectively.

The crystal structure of MetRsBm (4DLP) was obtained from protein data bank (http://www.rcsb.org/pdb/explore.do? structureId=4DLP). The protein was prepared by removing of other chain, waters, and heteroatoms, by adding hydrogen, and energy minimized at 0.30 Å RMSD using Prot-Prep module. The glide module is built upon a grid-based algorithm that requires grid generation in the active site of the target protein. Then, A 10 × 10 × 10 Å grid was generated around on the active site of the target protein Tyr35, Asp72, Val250, Trp251, Asp253, Ala254, Leu255, Asn257, and Tyr258. The ligands were flexibly docked on the protein structure. The non-planar conformations were penalized. The ligands were having more than 200 atoms or more than 35 rotatable bonds were not docked. Also, the Van Der Waal's radius scaling factor was set to 0.8, and the partial charge cutoff was set to 0.15. In GLIDE docking, the top 10 compounds were selected based on extra precision G-score. The binding affinity of docked complexes was re-scored using X-Score v1.2.1 [13]. Protein-ligand interaction was analyzed by using Pymol version 1.1r. www.pymol.org and LigPlot+ v1.4.5.

## Results and Discussion

### Docking analysis of known inhibitors of MetRSBm

The molecular docking of known inhibitors of MetRSBm was done using glide module. All three known inhibitors showed Gscore from -3.88 to -3.17 kcal/mol and predicted binding energy from -8.66 to -8.30 kcal/mol (calculated using the X-Score) (Table 1). The Ligplot+ analysis showed that His44, Lys77, Gln173, and Trp251 amino acids interact by h-bond interaction, with docked ligands. These results suggest that the novel MetRSBm inhibitors could be designed considering parameters of docking results leading to new potent drugs against Brucella.

### Screening of ChEMBL antimycobacterial library against MetRSBm

**Figure 1:**
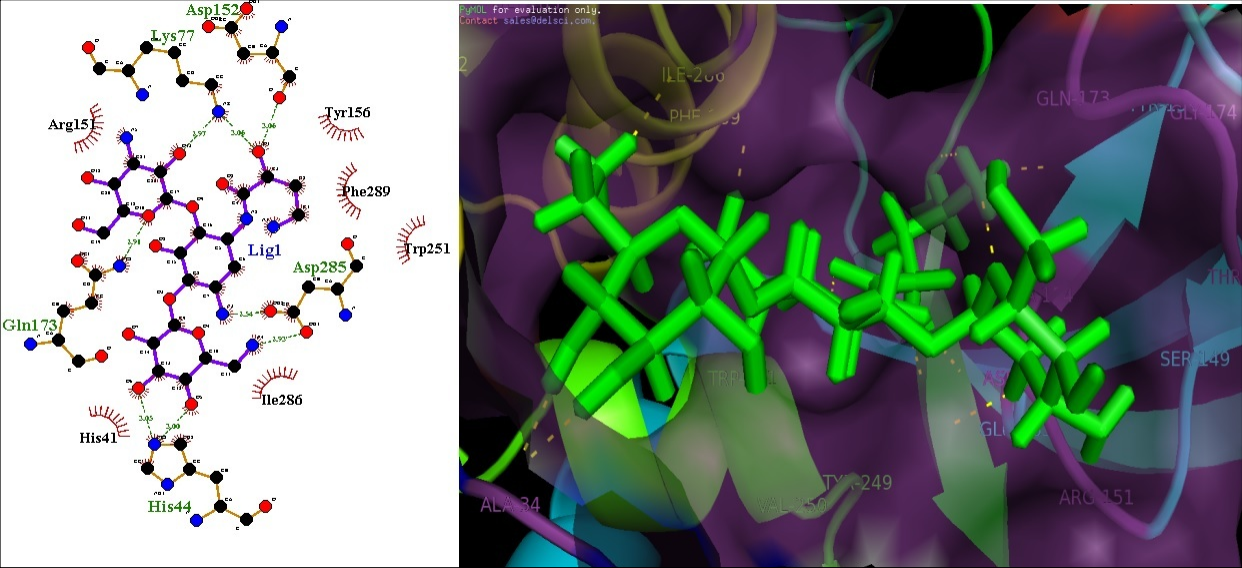
Inhibitor ligand CHEMBL177 or Amikacin (ChEMBL antimycobacterial dataset) bound to the active sites of the MetRSBm. (A) Details of MetRSBm-ligand interaction. Key residues within 5.0 sphere of top-ranked in the binding pocket are shown. (B) Purple colour molecular surface shows the active site cleft in which compound ligand binds.

ChEMBL antimycobacterial dataset (56400) was subjected to molecular docking. The top 10 compounds (after docking), based on their G-score are shown in Table 2. The glide score of these compounds varies from -10.27 to -8.42 (in kcal/mol). The G-score indicated that these compounds have a good binding affinity for MetRSBm enzyme. Figure 1 showed the docked complex of ligand Amikacin in the active site of the receptor with best Gscore (-12.27 kcal/mol). To further validate in silico, predicted binding affinity of the best pose obtained from docking studies for each compound was calculated using X-score program was found to be in between -9.27 and -7.25 kcal/mol shown in Table 2.

We analyzed the types of interactions of each top ranked ChEMBL antimycobacterial compound against MetRSBm; 2D plots were generated using Ligplot+ software and ligand-protein complex. The number of hydrogen bonded interactions, lipophilic interactions and the number of non-bonded interactions was counted and tabulated in Table 2. It is observed that overall all compounds from C1 to C10 have formed at least 1 (C2, C5, C6, C7, C9 and C10), mostly 5 (C1, and C4, and C8), and at most 7 (C3) hydrogen bonds. The total number of lipophilic interactions for each compound varies in between 15 (for C5) and 5 (for C1). Also, the total number of non-bonded interactions for each compound varies from 74 (for C5) to 29 (for C2). These observations suggest that the compounds C1, C3, C4, and C6 have better specificity as they have more hydrogen bonds and compounds C1, C3, C4, C5, C8 and C9 have good binding affinity due to a high number of hydrophobic contacts. The Compound C1 showed interaction with Glide score -12.27 kcal/mol. The docking poses analysis of C1shows five hydrogen bonds (His44, Lys77, Asp15, Gln173, and Asp285) interaction with amino acid residues of the protein. The Compound C3 showed highest seven hydrogen bond interaction with the active site residues Ile33, Tyr35, Asn37, Lys77, Gln173, Tyr258, and Asp285, with Gscore of -9.56 kcal/mol, 56 nonbonded interactions, and six hydrophobic interactions (Ala32, Ala34, Trp251, Asn257, Leu255, and His290). Tyr35, His44, Tyr258, Asp285, and Ile286 are found to be the most conserved residues, which is present in at least 8 out of 10 compounds. Hence, based on the Docking analysis against MetRSBm inhibitors, we conclude that these compounds have a better affinity with MetRSBm enzyme, thus are novel potential candidate to develop drugs against Brucella.

Further, we also analyzed the interactions of approved drugs Library’s top ranked inhibitors against MetRSBm (Table 3). The highest X score of -8.40kcal/mol was obtained with the Hexoprenaline drug having four hydrogen bonds (Ile33, Tyr155, Lys225, and Tyr249) interaction with amino acid residues of the protein. The total number of lipophilic interaction for each compound varies in between 11 (C4 and C6) and 4 (for C10). The Compound C7 showed highest seven hydrogen bond interactions with the active site residues (Ile33, Tyr35, His41, His44, Asp72, and Arg151) have good specificity and C4 and C6 have a good binding affinity. Ala34 amino acid is present in 10 out of 10 compounds, and Gln173, Trp251, and Ile286 8 out of 10 compounds are found to be the most conserved residues. Hence, based on the comparison between known MetRSBm inhibitors (as control) and top ten potent drugs, we conclude that these compounds could bind to MetRSBm with better affinity, thus are the potential candidate to develop drugs against Brucella.

**Figure 2:**
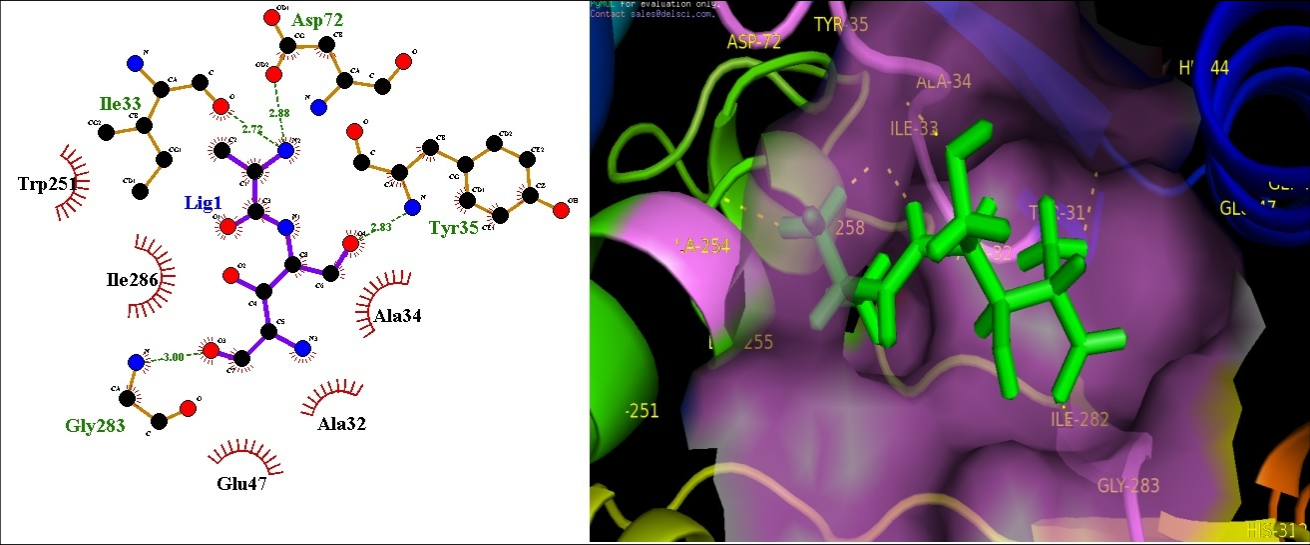
Inhibitor ligand Prumycin NSC278619 (Natural Product IV) bound to the active sites of the MetRSBm. (A) Details of MetRSBm-ligand interaction. Key residues within 5.0 sphere of top-ranked in the binding pocket are shown. (B) Magenta colour molecular surface shows the active site cleft in which compound ligand binds.

Additionally, we also analyzed the interactions of Natural Product IV Library’s top ranked inhibitors against MetRSBm (Table 4). Figure 2 shows the docked complex of ligand Prumycin in the active site of the receptor with best G-score (- 10.55 kcal/mol). The highest X score of -9.43 kcal/mol was obtained with the Chartreusin compound (C5) having two Hbond (Lys77, and Gln173) and G-Score -10.55 kcal/mol four Hbond (Ile33, Tyr35, Asp72, Gly283) interaction with amino acid residues of the protein. The total number of lipophilic interaction for each compound varies in between 14 (C6) and 4 (for C7). The Compound C1, C3, and C7 showed highest four hydrogen bond interactions with the active site residues have good specificity, and C6 have a good binding affinity. Ala32 is present in 10 out of 10 compounds, Trp251 and Ile286 are present in 9 out of 10 compounds, and Tyr35, Gln47 and Asp285 are found to be the most conserved residues, which is present in 8 out of 10 compounds. Hence, based on the comparison between known MetRSBm inhibitors (as control) and top ten potent drugs, we conclude that these compounds could bind to MetRSBm with better affinity, thus are the potential candidate to develop drugs against Brucella.

**Figure 3:**
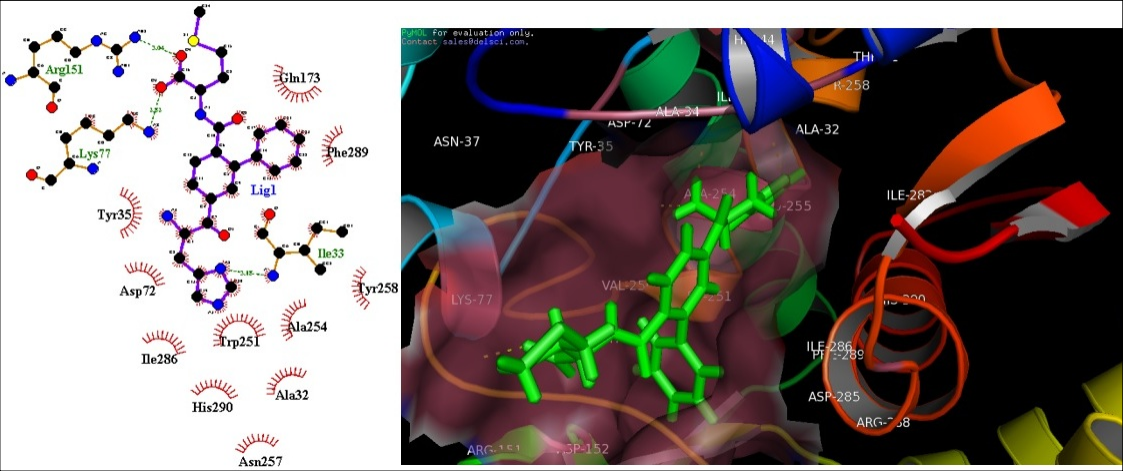
Inhibitor ligand CID69893052 (Methionine analogous) bound to the active sites of the MetRSBm. (A) Details of MetRSBmligand interaction. Key residues within 5.0 sphere of top-ranked in the binding pocket are shown; (B) ruby colour molecular surface shows the active site cleft in which compound ligand binds.

Interestingly, we also analyzed the interactions of methionine analogous Library’s top ranked inhibitors against MetRSBm (Table 5). Figure 3 shows the docked complex of ligand C1 compound in the active site of the receptor with the highest Gscore (-11.20) and X-score and -9.23 kcal/mol having two hydrogen bond (Ile33, and Arg151) interaction with amino acid residues of the protein. The total number of lipophilic interaction for each compound varies in between 14 (C7) and 7 (for C9). The Compound C2 and C9 showed highest seven hydrogen bond interaction with the active site residues have good specificity and C2, C4, C5, C6, C8, and C10 have a good binding affinity. Hence, based on the comparison between known MetRSBm inhibitors (as control) and top ten novel compounds, we conclude that these compounds could bind to MetRSBm with better affinity, thus are the potential candidate to develop drugs against Brucella.

## Conclusion

We extrapolate that the analogous of methionine for find novel drug likeness has been identified [4-(L-histidyl)-2-phenylbenzoyl] methionine hydrochloride, might show the inhibitor of Brucella melitensis effect by interacting with MetRS enzyme. In this study, we extrapolate that the analogous of methionine for find novel drug likeness has been identified [4-(L-histidyl)-2-phenylbenzoyl] methionine hydrochloride, might show the inhibitor of Brucella melitensis effect by interacting with MetRS enzyme. We suggests that Prumycin as a natural product is the novel drugs for brucellosis.

## Ethics approval and consent to participate

Not applicable

## Competing interests

The authors declare that they have no competing interests.

## Figures and Tables

**Table 1:** Molecular Docking results of the known inhibitors against MetRsBM

S. No.	Compound ID	IUPAC Name	G Score (kcal/mol)	X-Score (kcal/mol)	H Bond	Hydrophobic Interactions	No. of NB Interactions
1	1415, CID60195001	1-[3-[(3-chloro-5-methoxyphenyl)methylamino]propyl]-3-phenylurea	-3.88	-8.66	Gln173, Trp251	Ala32, Tyr35, Lys77, Ala154, Tyr156, Tyr249, Asp285, Ile286, Phe289	44
2	1433 CID60195274	1-[3-[(3,5-dichlorophenyl)methylamino]propyl]-3-thiophen-3-ylurea	-3.85	-8.03	Lys77, Trp251	Ala32, Ile33, Tyr35, Asp152, Ala154, Tyr156, Gln173, Tyr249, Phe289	33
3	1312, CID18353708	2-[3-[(3,5-dichlorophenyl)methylamino]propylamino]-1H-quinolin-4-one	-3.17	-8.3	His44, Lys77, Asp285,	Ala32, Tyr35, Glu47, Gln173, Trp251, Ile286, Phe289	30

**Table 2:** Top scoring 10 potential inhibitors from CHEMBL anti-myco-bacterial library against MetRsBM.

S. No.	Compound ID	IUPAC Name	G Score (in kcal/mol)	X-Score (kcal/mol)	H Bond	Hydrophobic Interactions	No. of NB Interactions
C1	CHEMBL177	(2S)-4-Amino-N-[(2S,3S,4R,5S)-5-amino-2-[(2S,3R,4S,5S,6R)-4-amino-3,5-dihydroxy-6-(hydroxymethyl)oxan-2-yl]oxy-4-[(2R,3R,4S,5R,6R)-6-(aminomethyl)-3,4,5-trihydroxy-oxan-2-yl]oxy-3-hydroxy-cyclohexyl]-2-hydroxybutanamide	-10.27	-7.91	His44, Lys77, Asp15, Gln173, Asp285	Arg151, Tyr156, Ile286, Trp251, Phe289	52
C2	CHEMBL235241	N'-(7-chloroquinolin-4-yl)propane-1,3-diamine	-9.6	-7.25	Ile33, Asp72	Ala34, Tyr35, Glu47, Trp251, Ala254, Asp285, Ile286	29
C3	CHEMBL471678	3-(2,3-dihydroxy-3-methylbutyl)-6-hydroxy-2-[(1E,5E)-3,4,10-trihydroxyundeca-1,5-dienyl]benzaldehyde	-9.56	-8.61	Ile33, Tyr35, Asn37, Lys77, Gln173, Tyr258, Asp285	Ala32, Ala34, Trp251, Leu255, Asn257, His290	56
C4	CHEMBL1644895	(2S)-N-[[(2R,3S,4R,5R)-5-(2,4-dioxo-1,3-diazinan-1-yl)-3,4-dihydroxyoxolan-2-yl]methyl]-3-(1H-imidazol-5-yl)-2-[[2-[(2R,3R,4R,5R,6R)-3,4,5-trihydroxy-6-(hydroxymethyl)oxan-2-yl]acetyl]amino]propanamide	-9.52	-8.49	Tyr35, His41, His44, Glu47, Lys77	Asp15, Ala32, Ala34, Asn37, Gly38, Gln173, Trp251, Ile286, Phe289	65
C5	CHEMBL2017735	3-[4-[(5-cyclopropyl-1H-pyrazol-3-yl)amino]-6-[2-(dimethylamino)ethylamino]pyrimidin-2-yl]benzonitrile	-9.29	-9.27	Asp72	Ile33, Tyr35, Gly43, Glu47, Lys77, Gln173, Trp251, Ala254, Leu255, Tyr258, Asp285, Ile286, His290, Phe314	74
C6	CHEMBL450837	N'-(7-chloroquinolin-4-yl)-N-propylethane-1,2-diamine	-8.6	-7.57	Ile33	Ala32, Ala34, Tyr35, His44, Glu47, Asp72, Trp251, Ala254, His290, Phe314	33
C7	CHEMBL240758	1-(4-bromophenyl)-3-imidazol-1-yl-2-(imidazol-1-ylmethyl)propan-1-one	-8.6	-7.68	Tyr35	Ala32, Ile33, Ala34, His44, Glu47, Asp72, Trp251, Gly283, Asp285, Ile286, His312, Phe314	41
C8	CHEMBL1801945	[(2R,3R,4S,5R,6R)-6-[[(3aS,7R,7aS)-7-hydroxy-4-oxo-1,3a,5,6,7,7a-hexahydroimidazo[4,5-c]pyridin-2-yl]amino]-5-[[(3S)-3-amino-6-[[(3S)-3,6-diaminohexanoyl]amino]hexanoyl]amino]-4-hydroxy-2-(hydroxymethyl)oxan-3-yl] carbamate	-8.52	-7.44	Tyr35, His44, Glu47, Gln173, Asp285	Asn37, His41, Asp152, Gly283, Ile286, His312, Phe314	53
C9	CHEMBL1200847	(4E)-4-[2-[4-(diaminomethylidene)cyclohexa-2,5-dien-1-ylidene]ethylidene]-3-oxocyclohexa-1,5-diene-1-carboximidamide	-8.45	-8.94	Asp152	Ala32, Ile33, Ala34, Tyr35, Glu47, Lys77, Gln173, Tyr251, Asp285, Ile286, Phe289,	50
C10	CHEMBL391443	1-(5-chlorothiophen-2-yl)-3-imidazol-1-yl-2-(imidazol-1-ylmethyl)propan-1-one	-8.42	-7.45	Tyr35,	Ala32, Ile33, Ala34, His44, Glu47, Asp72, Trp251, ly283, Asp285, Ile286, His312, Phe314	39

**Table 3:** Top scoring 10 potential inhibitors from Approved drugs library against MetRs-BM.

S. No.	Generic Name	G Score (in kcal/mol)	X-Score (kcal/mol)	H Bond	Hydrophobic Interactions	No. of NB Interactions
C1	Amikacin	-9.08	-7.86	His44, Lys77, Asp152, Gln173	Ala34, Tyr35, Arg151, Tyr156, Trp251, Asp285, Ile286, Phe289,	52
C2	adenosine triphosphate	-8.91	-7.17	Tyr35, Asn37, His44, Lys77, Trp251	Ala34, Asp152, Gln173, Asp285, Ile286, Phe289,	35
C3	Streptomycin	-7.53	-7.23	Tyr35, His44, Asp285,	Ala34, Gly43, Asp152, Gln173, Trp251, Ile286, Phe314	48
C4	Aztreonam	-7.29	-8.39	Asp72, Lys77	Ala32, Ile33, Ala34, Tyr35, His44, Glu47, Trp251, Ala254, Asp285, Ile286, His312	60
C5	Lymecycline	-7.06	-8.15	Ile33, Lys77, Asp152	Ala32, Ile33, Ala34, Tyr35, Asn37, His41, Arg151, Gln173, Thr175, Phe314,	38
C6	Hexoprenaline	-6.83	-8.4	Ile33, Tyr155, Lys225, Tyr249	Ala32, Ala34, Tyr35, Asp152, Ala154, Gln173, Phe219, Trp251, Ile286, Phe289, Phe314,	51
C7	Enviomycin	-6.72	-7.44	Ile33, Tyr35, His41, His44, Asp72, Arg151	Ala34, Asn37, Lys77, Gln173, Trp251, Asp258, Phe314,	43
C8	Fludarabine	-6.64	-7.11	Lys77, Asp152, Gln173, Trp251	Ala32, Ile33, Tyr35, Ile286, Phe289,	32
C9	Adenylate	-6.63	-7.14	Glu47, Gln173, Trp251, Asp285	Ala32, Ala34, Tyr35, Ile286, Phe289	31
C10	Paromomycin	-6.6	-7.21	Tyr35, His44, Lys77, Asp285	His41, Gln173, Ile286, Phe314	29

**Table 4:** Top scoring 10 potential inhibitors from natural product IV dataset against MetRs-BM.

S. No.	Compound ID	Generic Name	G Score (in kcal/mol)	X-Score (kcal/mol)	H Bond	Hydrophobic Interactions	No. of NB Interactions
C1	NSC 278619	Prumycin	-10.55	-6.74	Ile33, Tyr35, Asp72, Gly283	Ala34, Ala32, Glu47, Trp251, Ile286	36
C2	NSC 100858	d-Inositol Kasugamycin	-9.38	-7.62	Gln173, Trp251	Ile33, Tyr35, Gly43, His44, Glu47, Lys77, Asp285, Ile286, Phe289, Phe314	36
C3	NSC 256942	Epirubicin	-8.66	-8.71	His41, Glu47, Lys77, Gly283	Ala32, Ala34, His44, Asp152, Gln173, Trp251, Asp285, Ile286, Phe289, Phe314	61
C4	NSC 82151	Daunorubicin	-7.86	-9.24	Glu47	Ala32, Tyr35, Asn37, His41, His44, Lys77, Asp152, Gln173, Trp251, Gly283, Aps285, Ile286, Phe289, Phe314	55
C5	NSC 5159	Chartreusin	-7.27	-9.43	Lys77, Gln173	Ala32, Ile33, Ala34, Tyr35, Gly43, His44, Glu47, Arg151, Trp251, Aps285, Ile286,	57
C6	NSC 32944	(-)-Cephaeline, dihydrochloride	-7.22	-9	Lys77	Ala32, Ile33, Ala34, His41, Gly43, His44, Glu47, Asp152, Gln173, Trp251, Aps285, Ile286, Phe314	53
C7	NSC 2080	Melezitose	-6.95	-6.87	Tyr35, Asn37, Lys77, Gln173, Asp285	Ala32, Ala34, His44, Ile286	34
C8	NSC 105827	Thiosangivamycin	-6.28	-7.04	His44, Gln173, Trp251	Ala32, Tyr35, Lys77, Asp285, Ile286, Phe289	24
C9	NSC 407308	Sakuranin	-6.14	-8.57	Glu47, Asp152	Ala32, Ile33, Ala34, Tyr35, Gly43, His44, Lys77, Gln173, Trp251, Ile286, Phe289, His312, Phe314	54
C10	NSC 12865		-6	-7.46	Asp285	32, Ile33, Ala34, Tyr35, Trp251, Phe314	38

**Table 5:** Top scoring 10 potential inhibitors from Methionine analogus against MetRs-BM.

S. No.	Compound ID	G Score ( in kcal/mol)	X-Score (kcal/mol)	H Bond	Hydrophobic Interactions	No. of NB Interactions
C1	CID 69893052	-11.2	-9.23	Ile33, Arg151	Ala32, Tyr35, Asp72, Trp251, Ala254, Asn257, Tyr258, Ile286, Phe289, His290, Gln173	68
C2	CID 18244364	-11.03	-8.95	Ile33, Tyr35, Glu47, Asp72, Lys77, Arg151, Asp285	Ala32, Ala34, Asp152, Gln173, Gly174, Thr175, Trp251, Ala254, Phe289	82
C3	CID 19881009	-10.7	-8.27	Ile33, Tyr35, Glu47, Asp72, Lys77, Arg151, Gln173,	Ala32, Ala34, His44, Trp251, Gly283, Asp285, Ile286, His290, His312	61
C4	CID 71464640	-10.04	-7.5	Lys77, Asp285	Ala32, Ile33, Ala34, Tyr35, Gly38, Lys39, His41, Glu47, Asp72, Asp152, Gln173, Trp251, Ile286	65
C5	CID 2279	-10.04	-8.66	Lys77, Arg151	Ala32, Ile33, asp72, Asp152, Gln173, Trp251, Ala254, Tyr258, Asp285, Ile286, Phe289, His290	76
C6	CID 18408186	-9.94	-8.14	Ile33	Ala32, Ala34, Tyr35, His44, Glu47, Asp72, Trp251, Ala254, Leu255, Tyr258, Ile286, His290,	53
C7	CID 7005065	-8.91	-7.96	Ile33	Ala32, Ala34, Tyr35, His44, Glu47, Asp72, Trp251, Ala254, Leu255, Asn257, Tyr258, Ile286, His290, Phe314	47
C8	CID 54604718	-8.74	-7.93	Ile33, Tyr35, His290,	Ala34, His44, Asp72, Lys77, Gln173, Trp251, Ala254, Asn257, Tyr258, Asp285, Ile286, Phe289,	55
C9	CID 71402635	-8.63	-7.95	Ile33, Tyr35, Glu47, Asp72, Lys77, Gln173, Asp285	Ala32, Asn37, His44, Trp251, Ala254, Ile286, Phe314	58
C10	CID 44384676	-8.51	-7.66	Trp251, Asp285,	Ala32, Ile33, Ala34, Tyr35, Asp72, Lys77, Gln173, Ala254, Leu255, Tyr258, Phe289, His290,	66

## Abbreviations

MetRS: Methionyl-tRNA-Synthetase
MetRSBm: MethionyltRNA-Synthetase of Brucella melitensis
HTVS: High Throughput Virtual Screening
